# Tuning Optical Absorption and Device Performance in P3HT:PCBM Organic Solar Cells Using Annealed Silver Thin Films

**DOI:** 10.3390/polym18020254

**Published:** 2026-01-17

**Authors:** Alaa Y. Mahmoud

**Affiliations:** Department of Physical Sciences, College of Science, University of Jeddah, Jeddah 21589-80200, Saudi Arabia; aymahmoud8@uj.edu.sa

**Keywords:** organic solar cells, anodic interfacial layer, silver thin films, thermal annealing, device optimization, localized surface plasmon resonance

## Abstract

In this study, we investigated the effect of annealing ultrathin silver (Ag) films of varying thicknesses (1–6 nm) on both their optical absorption and the performance of poly(3-hexylthiophene-2,5-diyl) (P3HT) and [6,6]-phenyl-C_61_-butyric acid methyl ester (PCBM) organic solar cells (OSCs). The Ag films were deposited on indium tin oxide (ITO) anodes and annealed at 300 °C for 1–2 h to modify the anodic interface. The optical and electrical properties of the resulting devices were systematically characterized and optimized. The results revealed that a 1 nm AgO layer annealed for 2 h significantly enhanced the device performance, yielding a 6% increase in power conversion efficiency compared to the standard configuration. This improvement is attributed to two main factors: (i) a 25% increase in light absorption of the AgO/P3HT:PCBM film due to localized surface plasmon resonance of Ag nanoparticles and (ii) an 11% reduction in series resistance resulting from the favorable alignment of the Ag work function with the ITO anode and the polymer HOMO, which facilitates efficient hole extraction. These findings highlight the potential of ultrathin, annealed Ag/AgO interfacial layers as an effective strategy to enhance light absorption and charge transport in OSCs.

## 1. Introduction

**Figure 1:**
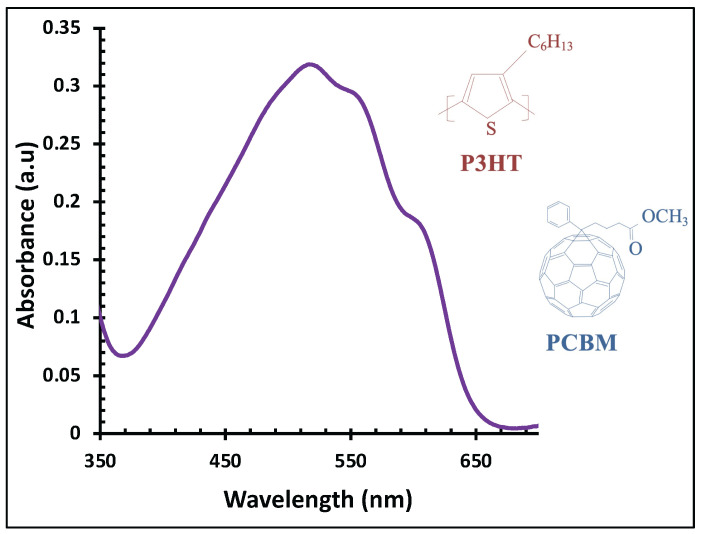
UV–visible absorbance spectrum of the P3HT:PCBM active layer, along with the chemical structures of P3HT and PCBM.

Harnessing solar energy through photovoltaic technologies has been a major focus of research for decades. Among these, organic solar cells (OSCs) have attracted considerable attention due to their unique advantages, including lightweight construction, mechanical flexibility, low-cost solution processability, and potential for large-area fabrication [1,2,3]. A particularly promising OSC architecture is the bulk heterojunction (BHJ), which forms a nanoscale interpenetrating network between electron-donating polymers and electron-accepting fullerene derivatives, facilitating efficient exciton dissociation and charge collection. Poly(3-hexylthiophene-2,5-diyl) (P3HT) and the fullerene derivative [6,6]-phenyl-C_61_-butyric acid methyl ester (PCBM) are among the most widely used materials for BHJ-OSCs. In a typical P3HT:PCBM device, the active layer is sandwiched between a transparent front electrode, such as indium tin oxide (ITO), and a metallic back electrode, such as aluminum (Al) [4,5,6]. Despite their advantages, P3HT:PCBM OSCs are generally limited to power conversion efficiencies below ~6% [7,8], constrained by factors such as interfacial chemical incompatibility [9,10], low open-circuit voltage [11,12,13], active layer degradation [14] and limitations in light absorption and layer thickness [4,15,16,17]. Figure 1 shows the chemical structures of P3HT:PCBM and its absorption spectrum, which exhibits a main absorption band from 420 to 620 nm, with a maximum at 520 nm and shoulders at 560 and 600 nm.

Various strategies have been explored to enhance OSC performance. These include introducing noble metal nanoparticles to improve light absorption [18,19,20,21,22], modifying cathodic interfaces to facilitate electron extraction [6,23,24,25], optimizing annealing conditions to enhance polymer:fullerene morphology [4,5,26], and engineering anodic interfacial layers to improve hole collection [27,28,29].

A major limitation in P3HT:PCBM OSCs is the restricted light absorption of the active layer. Increasing the thickness beyond ~250 nm to boost absorption is not practical, as it promotes charge recombination and increases series resistance (R_S_), both of which lower device efficiency [2,4,15,16,17,26,30,31]. Consequently, enhancing absorption without increasing thickness is critical, for example by integrating noble metal nanoparticles or ultrathin metallic films to exploit plasmonic effects [18,19,20,21,22].

Optimizing electrode interfaces is another key factor. Reducing R_S_ and facilitating charge extraction enhances PCE [9]. Techniques such as oxygen plasma treatment of ITO [32,33] and deposition of hole-transporting buffer layers like poly(3,4-ethylenedioxythiophene):poly(styrenesulfonate) (PEDOT:PSS) [34,35] improve interfacial quality. Deposition of noble metal or metal oxide thin films on the ITO surface can further tune the work function to promote efficient hole collection [1,9,19,36].

Among these materials, Ag/AgO thin films are promising for anodic interface modification due to their high transparency, conductivity, and absorptivity [37]. Prior studies have shown that annealing Ag nanofilms can significantly enhance their light absorption, which overlaps the P3HT:PCBM active layer spectrum [38,39,40]. However, there remains a research gap: while plasmonic and metal/metal-oxide interlayers have been explored for enhancing OSCs, the combined effect of Ag film thickness and annealing on both optical absorption and device performance in P3HT:PCBM OSCs has not been systematically investigated. Specifically, the relationship between ultrathin Ag/AgO interfacial layers, their localized surface plasmon resonance (LSPR)-enhanced absorption, energy-level alignment, and charge transport has not been fully elucidated.

In this work, we address this gap by studying the effect of annealing Ag nanofilms of varying thicknesses (1–6 nm) at 300 °C on their optical properties. We also examine how deposition and annealing of Ag films with thicknesses of 1, 2, and 6 nm influence the optical absorption, charge transport, and overall photovoltaic performance of P3HT:PCBM OSCs. This study provides insight into optimizing ultrathin Ag/AgO interfacial layers to enhance hole collection and device efficiency.

## 2. Materials and Fabrication

### 2.1. Chemical Materials

All chemicals and materials were used as received, with no additional purification. Regioregular poly(3-hexylthiophene) (P3HT, 98%) was sourced from Rieke Metals (Lincoln, NE, USA). The fullerene derivative [6,6]-phenyl-C_61_-butyric acid methyl ester (PCBM, >99.5%) and 1,2-dichlorobenzene (anhydrous, 99%) were purchased from Sigma-Aldrich (Burlington, MA, USA). Poly(3,4-ethylenedioxythiophene):poly(styrenesulfonate) (PEDOT:PSS, CLEVIOS™ P VP AI 4083), was obtained from HC Starck (Mayfield Heights, OH, USA). Silver (Ag), aluminum (Al), and lithium fluoride (LiF), each with a purity of 99.99% (metals basis), were supplied by Alfa Aesar (Thermos Fisher Scientific, Waltham, MA, USA).

### 2.2. Instrumentation

UV–visible absorption spectra were recorded using a double-beam spectrophotometer (PerkinElmer LAMBDA 650, PerkinElmer, Shelton, CT, USA) over the wavelength range of approximately 300–850 nm. The photovoltaic performance of the organic solar cell devices was evaluated under simulated solar illumination using a xenon-lamp solar simulator (Oriel Instruments) equipped with an AM 1.5G filter and operated under ambient conditions. The light intensity was calibrated to 100 mW cm^−2^ using a calibrated light meter (LI-250, LI-COR Biosciences, Lincoln, NE, USA). Current–voltage (J–V) characteristics were measured with a source-measure unit/electrometer (Keithley 2400, Keithley Instruments, Solon, OH, USA). Thin films of the active layer (P3HT:PCBM) were deposited using a spin coater (Laurell WS-650Mz-23NPPB, Laurell Technologies Corporation, Lansdale, PA, USA). Metal electrodes (Ag, Li, and Al) were deposited by thermal evaporation using a vacuum evaporator (Tecuum VCM 600 V1, Tecuum AG, Winterthur, Switzerland). In addition, a hotplate, ultrasonic bath, and plasma etcher were employed during the fabrication of the organic solar cells.

### 2.3. Fabrication of OSC Device

**Figure 2:**
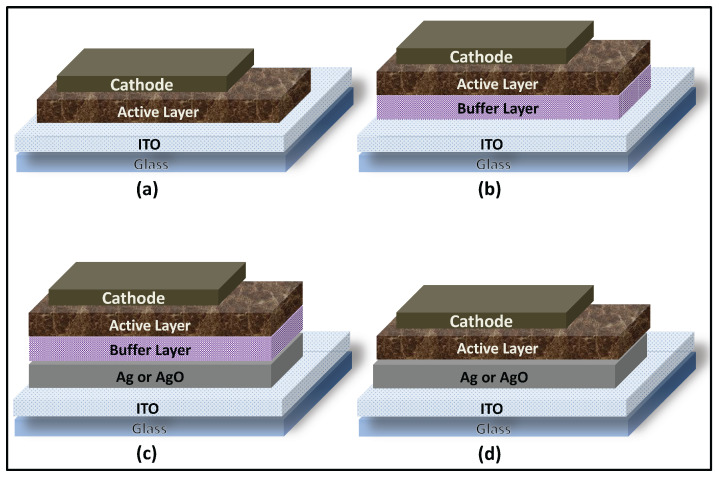
Schematic representations of the final device architectures: (**a**) device without a Ag nanofilm and without a buffer layer; (**b**) device without a Ag nanofilm but with a buffer layer; (**c**) device incorporating both a Ag nanofilm and a buffer layer; and (**d**) device incorporating a Ag nanofilm without a buffer layer. Ag refers to the silver film prior to annealing, while AgO denotes the same film after thermal annealing, during which it becomes oxidized.

To prepare the device substrates, indium tin oxide (ITO)-coated glass was cut to the desired dimensions, patterned, and initially cleaned with a laboratory detergent. The substrates were subsequently sonicated in acetone, isopropanol, and deionized (DI) water for 10 min each. After cleaning, the substrates were dried under a nitrogen stream, thermally treated at 150 °C for 20 min, and then exposed to oxygen plasma to improve surface cleanliness and wettability. In all devices, the ITO layer served as the anodic electrode. Standard devices with the architecture Glass/Anode/BufferLayer/ActiveLayer/Cathode, as illustrated in Figure 2b, were fabricated as follows: An aqueous dispersion of the buffer layer material, PEDOT:PSS, was filtered through a 0.45 µm PVDF membrane and spin-coated onto the pretreated ITO substrates at 4000 rpm for 30 s, using an acceleration time of 10 s to reach 1100 rpm. The resulting films were annealed at 120 °C for 1 h to ensure complete solvent removal and subsequently transferred into a nitrogen-filled glove box for deposition of the active layer. The bulk heterojunction active layer was prepared by dissolving P3HT and PCBM (20 mg mL^−1^, 1:0.8 *w*/*w*) in 1,2-dichlorobenzene, followed by filtration through a 0.45 µm PTFE membrane. The solution was deposited onto the ITO/PEDOT:PSS stack using a three-step spin-coating process (200 rpm for 5 s, 500 rpm for 5 s, and 1000 rpm for 60 s) to form a uniform active layer. The as-cast films were placed in covered Petri dishes inside the glove box and allowed to dry under ambient conditions for 30 min.

Finally, the cathode was deposited by thermal evaporation under high-vacuum conditions (<10^−6^ Torr), forming a bilayer consisting of lithium fluoride (LiF, ~6 nm) and aluminum (Al, ~90 nm). Deposition rates were maintained at approximately 1 Å s^−1^ for LiF and 2.5–5 Å s^−1^ for Al. A shadow mask was employed to define the electrode geometry. Each substrate contained eight identical devices, each with an active area of 0.16 cm^2^. The final device configurations are summarized in Figure 2.

For devices incorporating a Ag nanofilm, silver was thermally evaporated onto pre-cleaned ITO-coated glass substrates. The deposition was carried out at a controlled rate of approximately 2.5–5 Å s^−1^. Following deposition, the substrates were placed on a hotplate and thermally annealed at 300 °C for either 1 or 2 h prior to completing the deposition of the remaining device layers. The annealing temperature was selected for exploratory purposes rather than as a pre-optimized value. Ag nanofilms with nominal thicknesses ranging from 1 to 6 nm were fabricated. The optical absorption characteristics of the deposited Ag nanofilms were subsequently examined using a UV–visible spectrophotometer.

## 3. Measurements and Discussion

### 3.1. Optical Absorption of Ag Nanofilms

**Figure 3:**
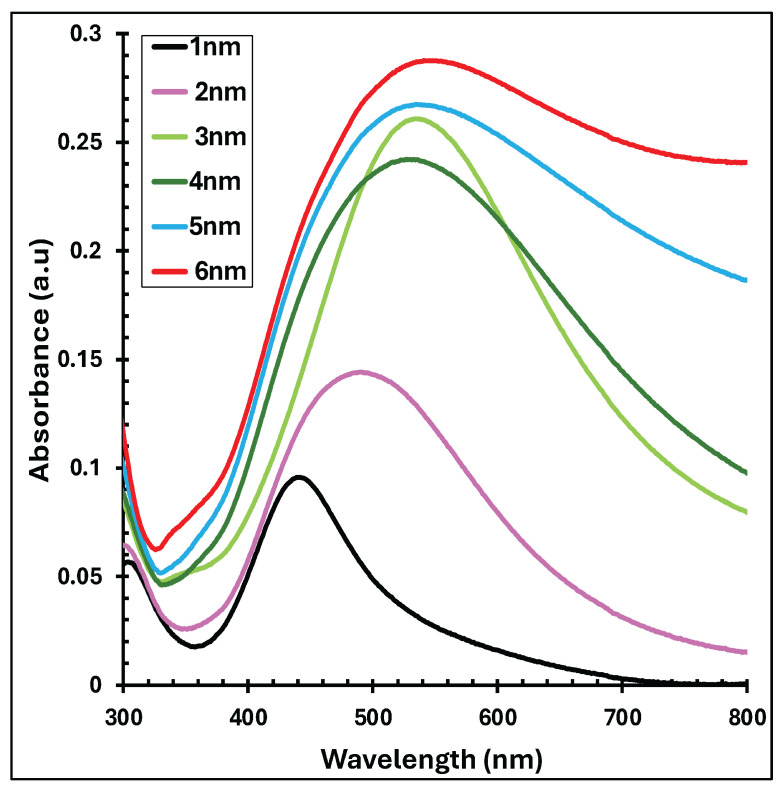
UV–visible absorbance spectra of thermally evaporated Ag nanofilms with nominal thicknesses ranging from 1 to 6 nm.

Figure 3 presents the UV–visible absorbance spectra of thermally evaporated Ag nanofilms with thicknesses ranging from 1 to 6 nm. As the Ag nanofilm thickness increases, a pronounced enhancement in absorbance intensity is observed. Concurrently, the characteristic absorption band becomes progressively broader and exhibits a red shift, moving from approximately 443 nm for the 1 nm Ag nanofilm to about 546 nm for the 6 nm film. These absorption features are attributed to LSPR, which arises from the collective oscillation of free electrons at the metal–dielectric interface, leading to strong light absorption and scattering in the visible region of the spectrum [41,42]. Notably, the spectral range of Ag nanofilm absorption significantly overlaps with that of the P3HT:PCBM active layer which is illustrated in Figure 1, indicating potential optical interaction between the plasmonic layer and the photoactive material.

### 3.2. Optical Absorption of Annealed Ag Nanofilms

**Figure 4:**
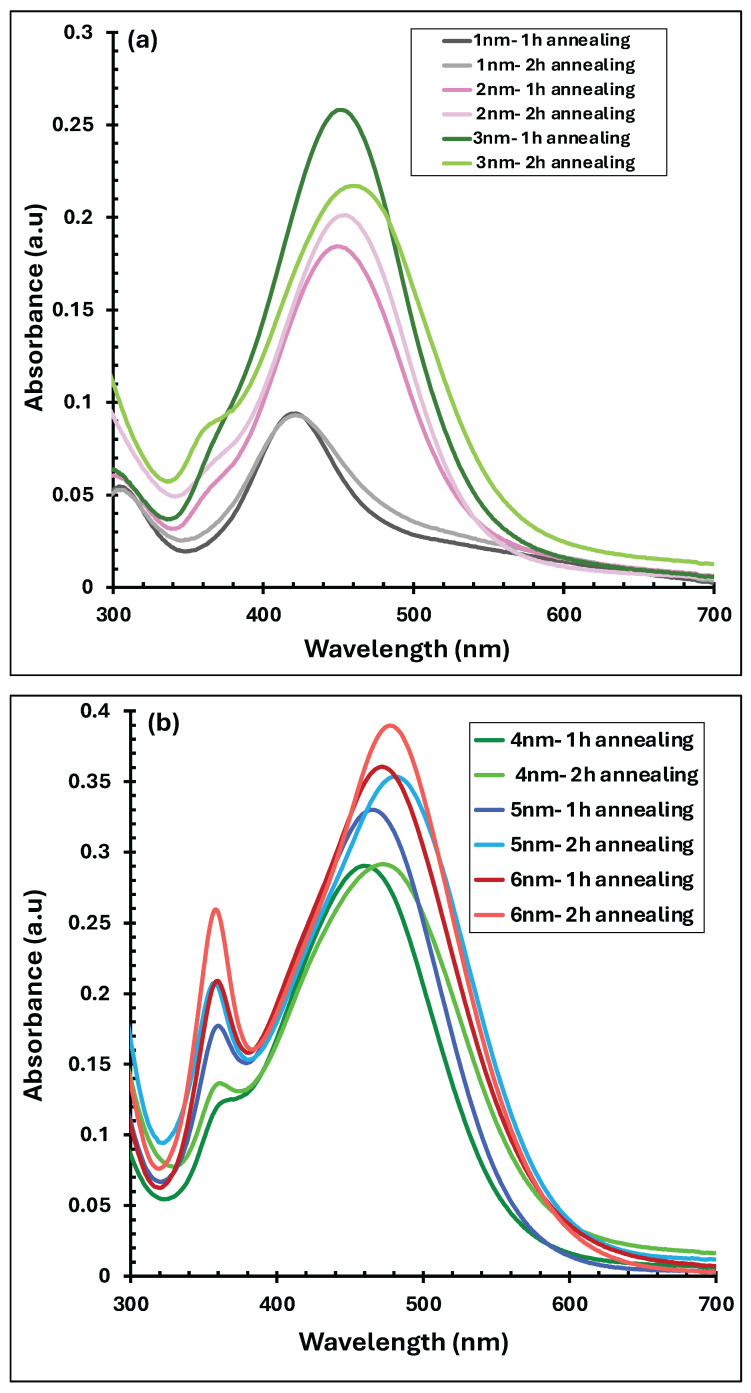
UV–visible absorbance spectra of thermally evaporated Ag nanofilms with thicknesses ranging from (**a**) 1–3 and (**b**) 4–6 nm, measured before and after annealing at 300 °C for 1 and 2 h.

Figure 4 compares the UV–visible absorption spectra of Ag nanofilms before and after thermal annealing at 300 °C for durations of 1 and 2 h. For the 1 nm Ag film, represented by the gray curves in Figure 4a, annealing did not significantly alter the absorption intensity; however, a noticeable blue shift in the plasmonic peak from approximately 445 nm to 421 nm was observed after annealing. In the case of the 2 nm Ag film (pink curves in Figure 4a), thermal treatment led to a clear enhancement in absorption intensity, particularly after 2 h of annealing, accompanied by a pronounced blue shift from about 500 nm to 421 nm. For the 3 nm Ag film (green curves in Figure 4a), annealing for 2 h resulted in a reduction in absorption intensity, while still inducing a blue shift in the absorption peak from approximately 540 nm to 450 nm. In contrast, thicker Ag nanofilms with nominal thicknesses of 4, 5, and 6 nm (yellow, blue, and red curves in Figure 4b, respectively) exhibited an overall increase in absorption intensity after annealing, with the most pronounced enhancement observed for the 2 h annealed samples. For these films, the main absorption peak shifted from around 550 nm to approximately 480 nm following annealing. In addition, secondary absorption peaks near 360 nm emerged for the 4–6 nm films after thermal treatment.

These results demonstrate that the optical absorption of Ag nanofilms is strongly influenced by annealing temperature, annealing duration, and initial film thickness. The enhanced absorption observed after annealing is attributed to the transformation of the continuous Ag nanofilm into discrete or quasi-spherical Ag nanoparticles, which support LSPR. Both the annealing temperature and time play critical roles in tuning the nanoparticle size and crystallinity, thereby modifying the position and intensity of the plasmonic absorption peaks [37,40]. The observed blue shifts in the absorption maxima after annealing are commonly associated with quantum confinement effects, suggesting a reduction in the effective particle size [39,43]. Furthermore, the appearance of secondary absorption peaks in the annealed 4–6 nm films may be related to particle shape anisotropy or elongation, which enables plasmon excitation along multiple axes [1]. It should also be noted that annealing Ag nanofilms under ambient conditions can promote partial oxidation, leading to the formation of AgO due to increased atomic mobility and oxygen availability during air annealing [38,44].

### 3.3. Effect of 1 nm Ag/AgO Film on OSC Parameters

A 1 nm Ag layer was thermally evaporated onto pre-cleaned ITO-coated glass substrates, which were subsequently annealed at 300 °C for 2 h prior to deposition of the remaining device layers. For comparison, four sets of devices, corresponding to the architectures shown in Figure 2, were fabricated simultaneously: (1) ITO/Active Layer/Cathode, (2) ITO/BufferLayer/ActiveLayer/Cathode, (3) ITO/1 nm AgO (2 h-annealing)/BufferLayer/ActiveLayer/Cathode, (4) ITO/1 nm AgO (2 h-annealing)/ActiveLayer/Cathode. To evaluate the effect of the Ag/AgO layer on hole collection efficiency and overall device performance, the influence of the Ag/AgO films on both the optical and electrical properties of the devices was investigated.

**Figure 5:**
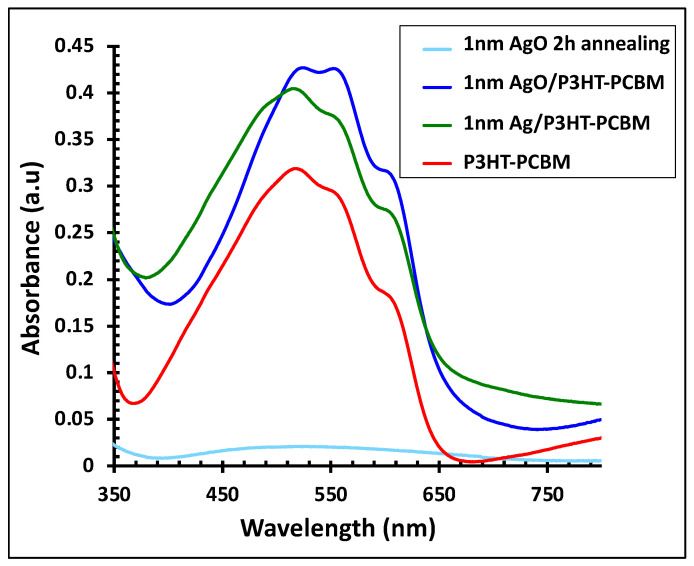
UV–visible absorbance spectra of the 1 nm Ag/P3HT:PCBM film before and after annealing at 300 °C for 2 h, compared with the pristine P3HT:PCBM layer and the 1 nm AgO film after 2 h of annealing.

Figure 5 displays the absorption spectra of the P3HT:PCBM active layer alone and in combination with a 1 nm Ag film, before and after 2 h of annealing. The faint blue line represents the absorption of the AgO film after annealing. Incorporation of the Ag nanofilm into the P3HT:PCBM layer (green line) enhanced the light absorption intensity by approximately 25% and broadened the absorption range from 420–620 nm to 380–650 nm. Following 2 h of annealing, the resulting AgO/P3HT:PCBM composite exhibited a further increase in absorption intensity (~34%) and a red shift in the absorption peak, indicating improved light-harvesting capabilities.

**Figure 6:**
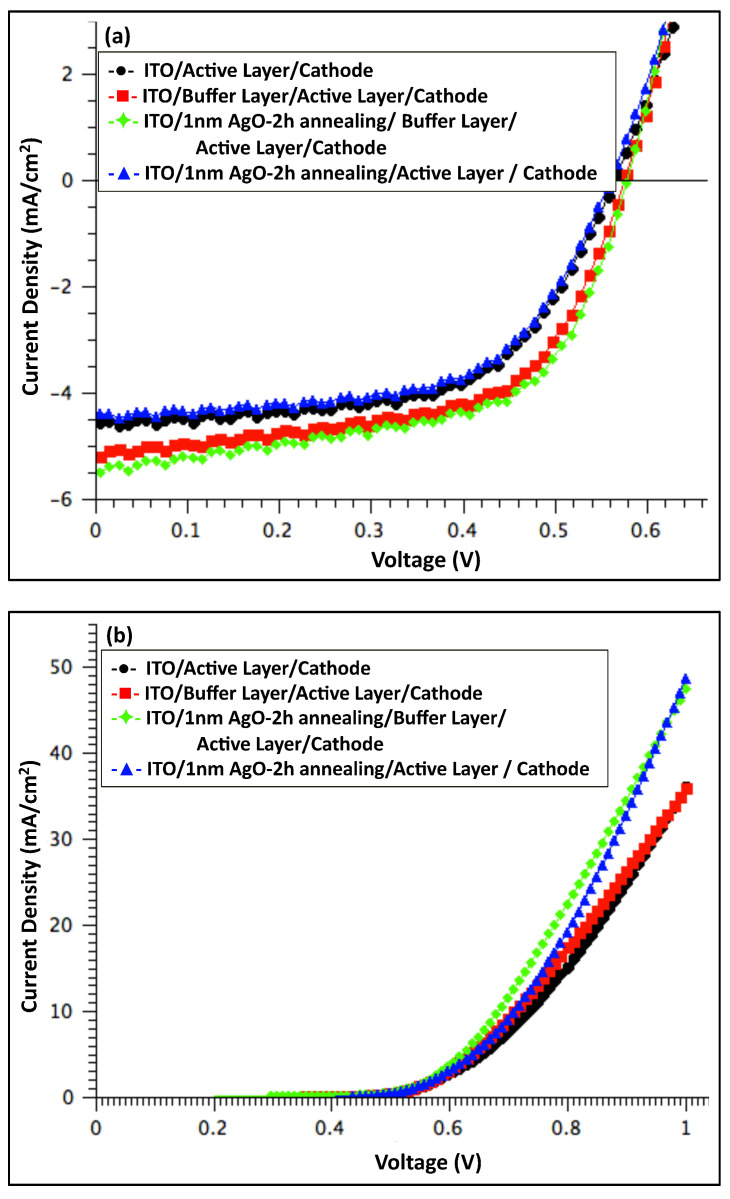
J–V characteristics of organic solar cell devices: comparison between devices with a 1 nm Ag film annealed at 300 °C for 2 h, devices without Ag, and devices without both Ag and buffer layers, measured (**a**) under illumination and (**b**) in the dark.

Figure 6 shows the J–V characteristics of organic solar cell devices measured under illumination (a) and in the dark (b), comparing devices before and after incorporation of a 1 nm Ag/AgO film (pristine and after 2 h of annealing). And Table 1 summarizes the average performance of photovoltaic parameters, including open-circuit voltage (V_OC_), short-circuit current density (J_SC_), fill factor (FF), and power conversion efficiency (PCE). The series resistance (R_S_) was estimated from the slope of the dark I–V curves at 0.9 V.

The photovoltaic performance was optimized in devices featuring a modified anode consisting of a buffer layer combined with a 1 nm AgO film annealed at 300 °C for 2 h. These devices exhibited a PCE of 1.9%, representing a 6% improvement compared to the reference. This enhancement primarily arose from a 6% increase in the J_SC_, rising from 5.20 to 5.51 mA cm^−2^, and an 11% reduction in R_S_. The V_OC_ remained unchanged relative to the pristine device. The simultaneous increase in J_SC_ and decrease in R_S_ indicate improved charge collection and overall device performance.

### 3.4. Effect of 2 nm Ag Film on OSC Parameters

In this set of experiments, a 2 nm Ag layer was thermally evaporated onto pre-cleaned ITO substrates and subsequently annealed at 300 °C for 2 h prior to deposition of the remaining device layers. Four sets of devices were fabricated simultaneously to evaluate the impact of the Ag/AgO layer on device performance: (1) ITO/BufferLayer/ActiveLayer/Cathode, (2) ITO/2 nm Ag/BufferLayer/ActiveLayer/Cathode, (3) ITO/2 nm AgO (2 h-annealing)/BufferLayer/ActiveLayer/Cathode, (4) ITO/2 nm AgO (2 h-annealing)/ActiveLayer/Cathode.

**Figure 7:**
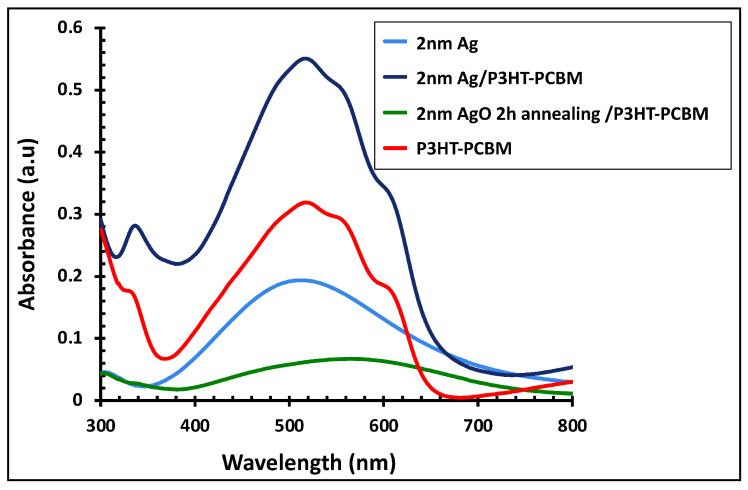
UV–visible absorbance spectra of the 2 nm Ag/P3HT:PCBM film before and after annealing at 300 °C for 2 h, compared with the pristine P3HT:PCBM active layer and the 2 nm Ag film alone.

Figure 7 displays the absorption spectra of the P3HT:PCBM active layer alone and in combination with the 2 nm Ag film, both before and after 2 h of annealing. The blue line represents the as-deposited 2 nm Ag film without annealing. Incorporation of the Ag nanofilm into the P3HT:PCBM layer, shown by the dark line, significantly enhanced the light absorption intensity by approximately 72% and extended the absorption range from 420–620 nm to 420–670 nm. In contrast, annealing the 2 nm Ag film for 2 h produced an AgO/P3HT:PCBM composite with markedly reduced absorption intensity and a red-shifted peak, altering the original absorption profile of the active layer.

**Figure 8:**
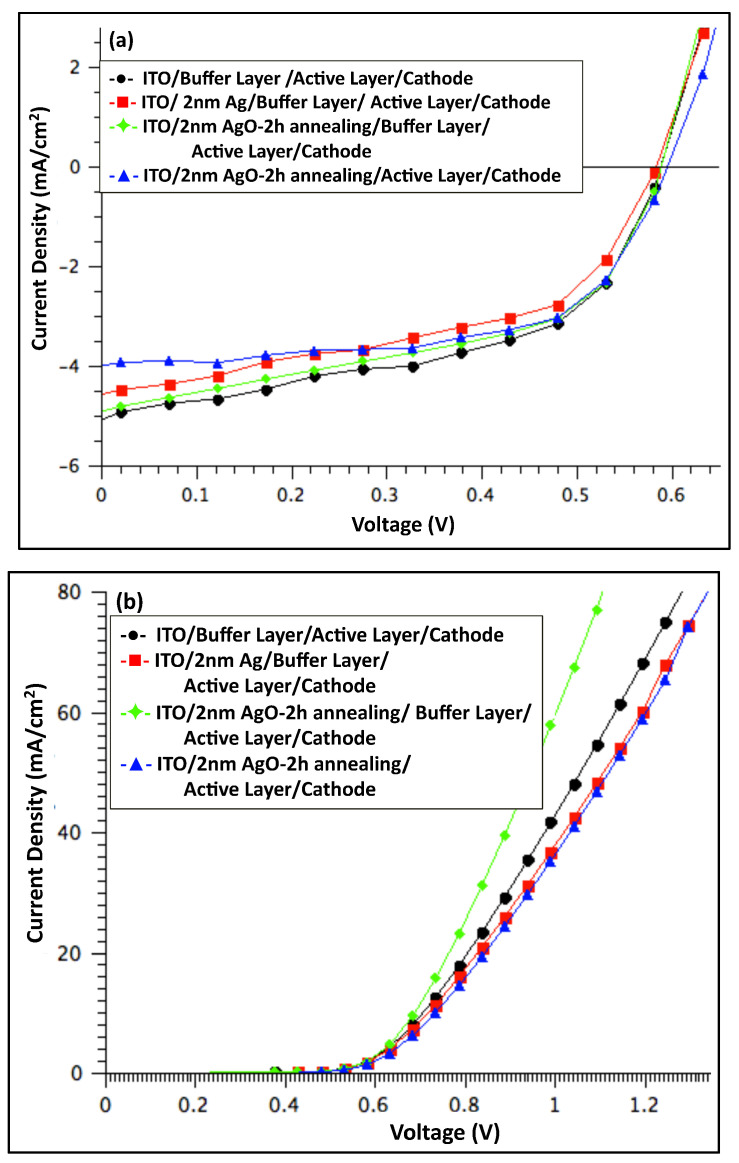
The J–V characteristics for OSC devices with and without 2 nm Ag annealed at 300 °C for 2 h, along with devices without a buffer layer or devices without annealing of Ag (**a**) under light and (**b**) in dark.

Figure 8 presents the J–V characteristics of organic solar cell devices measured (a) under illumination and (b) in the dark, comparing devices before and after incorporation of a 2 nm Ag/AgO film (pristine and after 2 h of annealing). Table 2 summarizes the average performance of photovoltaic parameters, including V_OC_, J_SC_, FF, and PCE. The R_S_ was determined from the slope of the dark I–V curves at 0.9 V.

The data in Table 2 indicate that adding a 2 nm Ag or AgO layer to the anodic interface negatively affected overall device performance. While the V_OC_ remained largely unchanged across all devices, the J_SC_ decreased by 2.2% for devices containing the 2 h annealed AgO film and by 9.3% for devices with the non-annealed Ag film. The highest performance was observed in devices without any modifications to the anodic buffer layer, suggesting that the absence of Ag or AgO nanofilms facilitates more efficient hole collection.

### 3.5. Effect of 6 nm Ag Film on OSC Parameters

In this experiment, a 6 nm Ag layer was thermally evaporated onto pre-cleaned ITO substrates and subsequently annealed at 300 °C for 1 and 2 h prior to deposition of the remaining device layers. For comparison, four sets of devices, corresponding to the architectures illustrated in Figure 2, were fabricated simultaneously: (1) ITO/BufferLayer/ActiveLayer/Cathode, (2) ITO/6 nm Ag/BufferLayer/ActiveLayer/Cathode, (3) ITO/6 nm AgO (1 h-annealing)/BufferLayer/ActiveLayer/Cathode, (4) ITO/6 nm AgO (2 h-annealing)/BufferLayer/ActiveLayer/Cathode. The impact of the Ag/AgO layer on hole collection efficiency was assessed by evaluating the overall performance of the devices.

**Figure 9:**
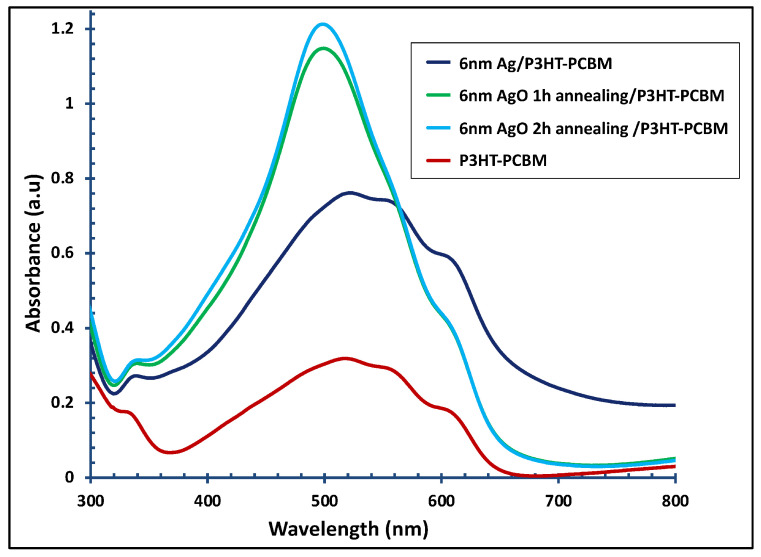
UV–visible absorbance spectra of the 6 nm Ag/P3HT:PCBM film before and after annealing at 300 °C for 1 and 2 h, compared with the pristine P3HT:PCBM active layer.

Figure 9 shows the UV–visible absorption spectra of the pristine P3HT:PCBM layer and the P3HT:PCBM layer combined with a 6 nm Ag film, both before and after annealing for 1 and 2 h. Incorporation of the 6 nm Ag film into the P3HT:PCBM layer (dark blue line) enhanced the absorption intensity by approximately 137% and broadened and red-shifted the absorption band from 420–620 nm to 420–670 nm. Thermal annealing of the Ag films for 1 and 2 h (green and faint blue lines) further modified the absorption profile of the P3HT:PCBM layer, resulting in a single, intense peak with higher absorptivity.

**Figure 10:**
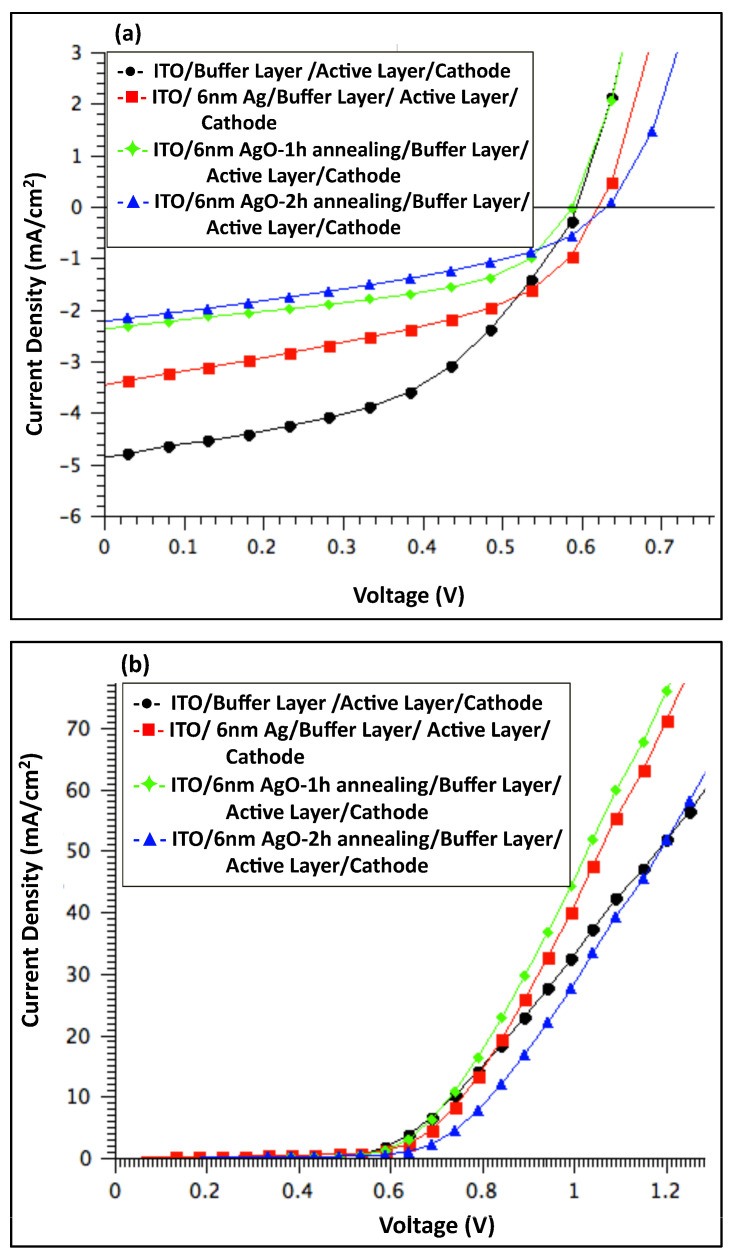
J–V characteristics of OSC devices with and without a 6 nm Ag film, including films annealed at 300 °C for 1 and 2 h, as well as devices without Ag or without annealing, measured (**a**) under illumination and (**b**) in the dark.

Figure 10 presents the J–V characteristics of organic solar cell devices measured (a) under illumination and (b) in the dark, comparing devices before and after incorporation of a 6 nm Ag/AgO film (pristine and after 2 h of annealing).

The average performances of photovoltaic parameters of devices incorporating a 6 nm Ag or AgO film are summarized in Table 3. The highest performance was achieved by the pristine device with the architecture ITO/Buffer Layer/Active Layer/Cathode. Introduction of the 6 nm Ag film led to a significant reduction in overall device performance, with a 47% decrease in PCE, primarily due to a 37% drop in the J_SC_. This indicates less efficient hole extraction by the anode compared to the pristine device. Annealing the Ag film further exacerbated this effect, resulting in a 60% reduction in J_SC_, likely due to the formation of energy-quenching states that limit charge carrier collection at the electrodes [45].

**Figure 11:**
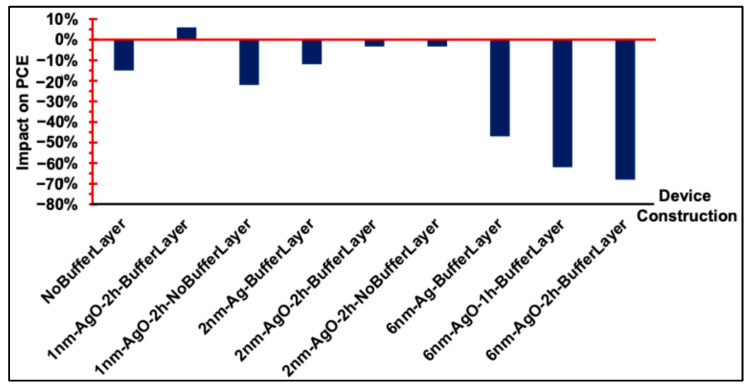
Comparison of the relative changes in PCE resulting from the incorporation of Ag or AgO interfacial layers in OSC devices.

The dark J–V characteristics of all fabricated devices, shown in Figure 6b, Figure 8b and Figure 10b, demonstrate good diode behavior. Under illumination, the performance of OSC devices with and without a PEDOT:PSS buffer layer and Ag/AgO interfacial layers is summarized in Figure 11. The results indicate that devices incorporating a PEDOT:PSS buffer layer consistently exhibited the best overall performance, serving as the standard configuration. Introducing an interfacial Ag or AgO layer to modify the anodic buffer primarily influenced the J_SC_ and R_S_, while the V_OC_ remained largely unaffected. Among the various modifications, a 1 nm AgO layer annealed at 300 °C for 2 h provided the most effective enhancement of the anodic buffer, improving charge collection and device performance.

**Figure 12:**
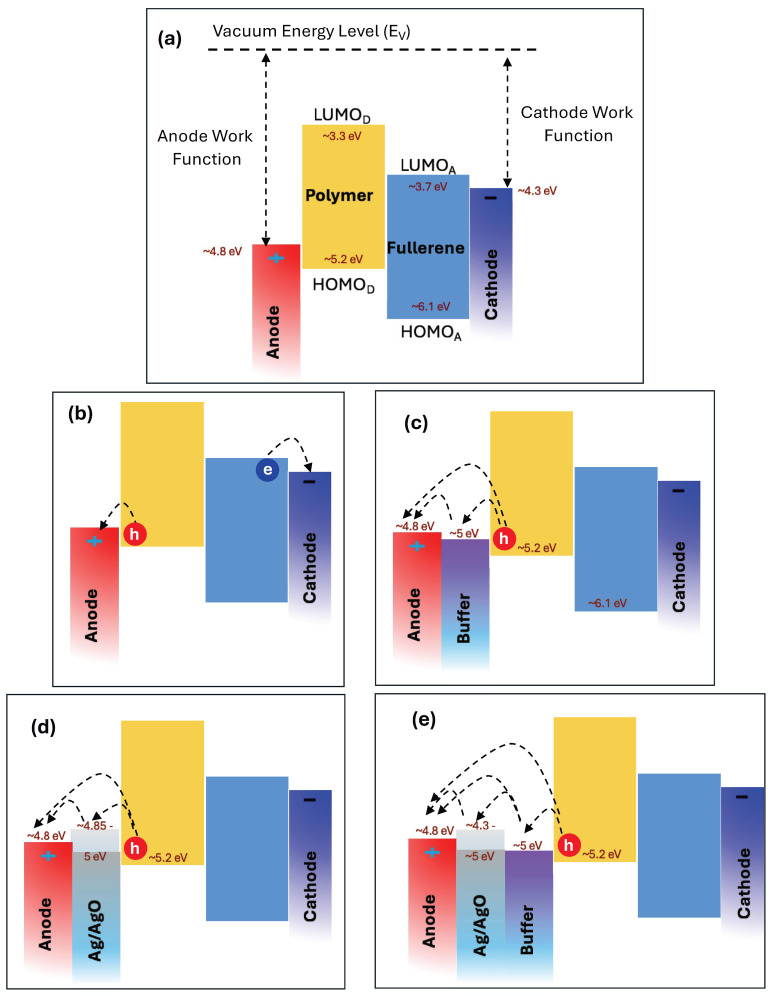
(**a**) Energy band diagram of the materials used in this study. (**b**) Schematic of charge carrier (electron and hole) transport to the respective electrodes. (**c**) Role of the buffer layer in facilitating hole collection. (**d**) Illustration of the Ag/AgO interfacial layer with varying work functions, showing optimal alignment with the polymer HOMO and the anode work function. (**e**) Energy band diagram and hole transport pathway for the OSC configuration exhibiting the best performance.

To explain these results, Figure 12 presents the energy band diagram of the materials employed in this study. The work functions of the electrodes, along with the HOMO and LUMO energy levels of both the polymer and fullerene, were obtained from reference [1]. The work function of Ag/AgO is dependent on both film thickness and annealing temperature, ranging from 4.3 to 5.0 eV [45,46,47]. As illustrated in Figure 12c–e, the Ag/AgO work function aligns well with the HOMO level of the polymer and the work function of the ITO anode, establishing an efficient hole-collecting interface. Notably, a 1 nm AgO film annealed at 300 °C for 2 h resulted in a 6% increase in PCE. This improvement can be attributed to several factors: (1) SPR of the Ag thin film enhances the light absorption of the P3HT:PCBM active layer (Figure 5), generating more charge carriers, increasing J_SC_, and boosting the PCE [1,48]; (2) Energy-level alignment between the AgO work function, the polymer HOMO, and the anode work function facilitates efficient hole transport to the anode, enhancing device performance; (3) Reduced sheet resistance and improved surface morphology of the AgO-modified layer—by approximately 11%—further support effective charge collection and transport. Together, these factors synergistically improve hole transport efficiency and overall photovoltaic performance in the OSC devices.

Increasing the thickness of the AgO layer and/or the annealing temperature resulted in devices with significantly reduced performance. This decline can be attributed to the oxidation behavior of silver, which is highly dependent on both annealing time and temperature. Excessive oxidation alters the grain size of the silver layer, adversely affecting charge transport and collection. Furthermore, the formation of an AgO oxide layer introduces energy-quenching states for charge carriers, limiting their mobility and reducing the photocurrent, ultimately diminishing the overall efficiency of the device [45].

## 4. Conclusions and Future Work

In this study, we systematically investigated the effect of thermally annealed Ag films of varying thicknesses (1–6 nm) at 300 °C on their optical absorption, as well as their influence on the performance of P3HT:PCBM-based organic solar cells. Silver thin films exhibited high transparency and conductivity, with absorption features overlapping the P3HT:PCBM active layer. Increasing the Ag film thickness led to enhanced absorption intensity, peak broadening, and a red shift, with the absorption maximum shifting from 443 nm for a 1 nm film to 546 nm for a 6 nm film. The improvement in light absorption after annealing was attributed to the formation of spherical Ag nanoparticles that support localized surface plasmon resonance. The annealing temperature and duration modulate the nanoparticle size, enhance crystallinity, and influence both absorption intensity and peak position.

The dark J–V curves of all fabricated devices indicated good diode behavior. Under illumination, devices incorporating a PEDOT:PSS buffer layer consistently exhibited the best performance. Among the various Ag/AgO interfacial modifications, a 1 nm AgO film annealed at 300 °C for 2 h provided the most effective enhancement of the anodic buffer layer, increasing the power conversion efficiency (PCE) by 6%. This improvement is attributed to the optimal alignment of the AgO work function with the polymer HOMO and the anode work function, which facilitates efficient hole transport to the electrode. In addition, the 1 nm AgO layer reduced the series resistance by 11% and improved surface morphology, both of which contribute to enhanced device performance. The LSPR effect of the Ag thin film further enhanced the active layer light absorption by ~25%, generating more charge carriers, increasing J_SC_, and boosting PCE.

However, increasing the AgO thickness or annealing time led to a deterioration in device performance. This decline is associated with accelerated silver oxidation, which alters grain size and reduces charge transport efficiency, and the formation of energy-quenching states that hinder free carrier collection, ultimately limiting photocurrent and overall device efficiency. Collectively, these findings demonstrate that ultrathin, optimally annealed AgO layers can serve as an effective anodic interfacial modification to improve OSC performance, while excessive thickness or annealing is detrimental.

Future research will focus on optimizing Ag/AgO nanostructures through precise deposition techniques to maximize LSPR effects while minimizing quenching. Alternative plasmonic materials, such as Au and hybrid interfacial layers combining metals with conductive polymers, could be explored to further enhance hole collection and reduce series resistance. Long-term stability studies under illumination and thermal stress are needed to assess practical device applicability. In addition, future work will include systematic imaging and chemical analysis to directly correlate Ag morphology, oxidation degree, series resistance, and charge-transport behavior with device performance. Conductivity and mobility measurements, which were not performed in this study, will also be incorporated to provide a more comprehensive understanding of the underlying mechanisms affecting device efficiency.

## Figures and Tables

**Table 1:** Photovoltaic parameters of OSC devices, for devices with a 1 nm Ag/AgO film annealed at 300 °C for 2 h, as well as for devices without Ag or buffer layers. The notation ±Δ denotes the change in the PCE value.

Device Configuration	V_OC_ (V)	J_SC_ (mA/cm^2^)	FF %	R_S_ (Ωcm^−2^)	PCE %	±Δ % PCE
ITO/BufferLayer/ActiveLayer/Cathode	0.58	−5.2	58.65	0.063	1.8	-
ITO/ActiveLayer/Cathode	0.57	−4.55	59.10	0.063	1.53	−15%
ITO/1 nm AgO-2 h Annealing /BufferLayer/ActiveLayer/Cathode	0.58	−5.51	58.48	0.056	1.9	+6%
ITO/1 nm AgO-2 h Annealing/Active Layer/Cathode	0.54	−4.25	59.19	0.063	1.4	−22%

**Table 2:** Photovoltaic parameters of OSC devices with and without a 2 nm Ag film (pristine and annealed at 300 °C for 2 h), including devices without a buffer layer. The notation ±Δ denotes the change in the PCE value.

Device Configuration	V_OC_ (V)	J_SC_ (mA/cm^2^)	FF %	R_S_ (Ωcm^−2^)	PCE %	±Δ % PCE
ITO/BufferLayer/ActiveLayer/Cathode	0.58	−4.92	52.67	0.056	1.51	-
ITO/2 nmAg/BufferLaye/ActiveLayer/Cathode	0.58	−4.46	51.19	0.056	1.33	−12%
ITO/2 nm AgO-2 hAnnealing/BufferLayer/Active Layer/Cathode	0.58	−4.81	52.21	0.038	1.46	−3.3%
ITO/2 nm AgO-2 hAnnealing/ActiveLayer/Cathode	0.58	−3.93	63.75	0.056	1.46	−3.3%

**Table 3:** Photovoltaic parameters of OSC devices with and without a 6 nm Ag/AgO film, including films annealed at 300 °C for 1 and 2 h, as well as devices without Ag annealing. R_S_ was determined from the slope of the dark I–V curve at 1.0 V. The notation ±Δ denotes the change in the value.

Device Configuration	V_OC_ (V)	J_SC_ (mA/cm^2^)	FF %	R_S_ (Ωcm^−2^)	PCE %	±Δ % PCE
ITO/BufferLayer/ActiveLayer/Cathode	0.59	−5.59	54.82	0.069	1.8	-
ITO/6 nmAg/BufferLayer/Active layer/Cathode	0.59	−3.51	46.25	0.044	0.95	−47%
ITO/6 nm AgO-1 h Annealing/BufferLayer/ActiveLayer/cathode	0.59	−2.37	48.73	0.041	0.68	−62%
ITO/6 nm AgO-2 h Annealing/BufferLayer/ActiveLayer/Cathode	0.64	−2.26	39.46	0.056	0.57	−68%

## Data Availability

The original contributions presented in this study are included in the article. Further inquiries can be directed to the corresponding author.
